# *ViReMa*: a virus recombination mapper of next-generation sequencing data characterizes diverse recombinant viral nucleic acids

**DOI:** 10.1093/gigascience/giad009

**Published:** 2023-03-20

**Authors:** Stephanea Sotcheff, Yiyang Zhou, Jason Yeung, Yan Sun, John E Johnson, Bruce E Torbett, Andrew L Routh

**Affiliations:** Department of Biochemistry and Molecular Biology, The University of Texas Medical Branch, Galveston, TX 77555, USA; Department of Biochemistry and Molecular Biology, The University of Texas Medical Branch, Galveston, TX 77555, USA; John Sealy School of Medicine, The University of Texas Medical Branch, Galveston, TX 77555, USA; Department of Microbiology and Immunology, The University of Rochester Medical Center, Rochester, NY 14642, USA; Department of Integrative Structural and Computational Biology, Scripps Research, La Jolla, CA 92037, USA; Department of Pediatrics, School of Medicine, University of Washington, Seattle, WA 98105, USA; Center for Immunity and Immunotherapies, Seattle Children's Research Institute, Seattle, WA 98105, USA; Institute for Stem Cell and Regenerative Medicine, University of Washington, Seattle, WA 98195, USA; Department of Biochemistry and Molecular Biology, The University of Texas Medical Branch, Galveston, TX 77555, USA; Sealy Center for Structural Biology and Molecular Biophysics, The University of Texas Medical Branch, Galveston, TX 77555, USA; Institute for Human Infections and Immunity, University of Texas Medical Branch, Galveston, TX 77555, USA

**Keywords:** next-generation sequencing, virus recombination, defective RNAs, defective viral genomes, copy-back RNAs

## Abstract

**Background:**

Genetic recombination is a tremendous source of intrahost diversity in viruses and is critical for their ability to rapidly adapt to new environments or fitness challenges. While viruses are routinely characterized using high-throughput sequencing techniques, characterizing the genetic products of recombination in next-generation sequencing data remains a challenge. Viral recombination events can be highly diverse and variable in nature, including simple duplications and deletions, or more complex events such as copy/snap-back recombination, intervirus or intersegment recombination, and insertions of host nucleic acids. Due to the variable mechanisms driving virus recombination and the different selection pressures acting on the progeny, recombination junctions rarely adhere to simple canonical sites or sequences. Furthermore, numerous different events may be present simultaneously in a viral population, yielding a complex mutational landscape.

**Findings:**

We have previously developed an algorithm called *ViReMa* (Virus Recombination Mapper) that bootstraps the *bowtie* short-read aligner to capture and annotate a wide range of recombinant species found within virus populations. Here, we have updated *ViReMa* to provide an “error density” function designed to accurately detect recombination events in the longer reads now routinely generated by the Illumina platforms and provide output reports for multiple types of recombinant species using standardized formats. We demonstrate the utility and flexibility of *ViReMa* in different settings to report deletion events in simulated data from Flock House virus, copy-back RNA species in Sendai viruses, short duplication events in HIV, and virus-to-host recombination in an archaeal DNA virus.

## Introduction

Recombination is essential for virus evolution and adaptation. Homologous recombination allows for the reshuffling of single-nucleotide variants (SNVs) among viral genomes or for the formation of chimeric viral genomes when they coinfect a single cell [1]. Such homologous recombination events have been attributed to the emergence of outbreak strains of viruses, including rhinoviruses and coronaviruses [2–4]. Recombination events that give rise to new and culturable strains/species of virus can readily be identified though phylogenetic comparison of full-length consensus genomes. Such studies inform on historical recombination events that gave rise to novel and “successful” emergent viruses that have survived selectivity filters.

Similarly, nonhomologous recombination in viral genomes can give rise to diverse genetic species. These range from simple insertions and deletions to more complex rearrangements of the virus genome, as well as intergenic recombination between different RNA virus species and/or their host. Deletion or insertion events can result in the formation of structural variants. For example, small 4 to 5 amino acid (AA) deletions are commonly seen surrounding the furin cleavage site of the severe acute respiratory syndrome coronavirus 2 (SARS-CoV-2) spike protein after cell culture passaging [5]. Larger structural variants have been reported that remove or disrupt entire viral genes, such as deletions in ORF 8 upon adaptation of SARS-CoV-1 to human transmission [6]. Simple duplications of the viral genome are frequently observed during the intrahost diversification of HIV during antiretroviral treatments. These duplications include short 9 to 21 AA stretches near the protease cleavage sites of GAG that result in altered processing kinetics of GAG by the HIV protease in the presence of protease inhibitors [7–11].

If the product of a nonhomologous recombination event is not a viable viral replicon, then the product is termed a “defective” viral genome (DVG) [12, 13] or “defective RNA.” Generally, DVGs are unable to produce the viral proteins required for replication, participle assembly, or other aspects of the viral replication cycle. Nevertheless, DVGs can still be replicated and passaged in *trans* by the parental or “helper” virus. DVGs sometimes have the ability to compete with or otherwise attenuate their parental “helper” viruses through a variety of different proposed mechanisms such as sequestration of cellular viral cofactor, competition with their helper viruses for access to the viral polymerase, or strong immunostimulation. Such DVGs are termed as “interfering.” While the presence and interference of DVGs have long been well appreciated in cell culture conditions [14], it is now emerging that the spontaneous emergence of DVGs during viral infections can have significant impacts on the outcomes of viral pathogenesis and vaccine design [15–17]. The term “defective” may therefore be somewhat misleading as it appears that such species may play an important role in the normal life cycle of some viruses.

Nonhomologous recombination may also give rise to intergenic recombination between 2 or more orthogonal templates. For example, in Flock House virus, a bipartite RNA virus from the *Nodaviridae* family, chimeric genetic species comprising fusion of the 2 genomic segments are spontaneously generated intracellularly during the replication cycle [18]. Intergenic recombination can also occur between the viral genomes and their hosts. For example, fragments of human ribosomal protein messenger RNAs (mRNAs) have been found to be integrated into the genomes of hepatitis E virus that were extracted from persistently infected patients. Interestingly, these virus–host chimeric strains displayed a growth advantage in cell culture [19, 20].

Next-generation sequencing (NGS) provides a high-throughput and quantitative tool to characterize the frequencies of different genetic products of viral recombination in complex virus populations. Canonical spliced or gapped sequence aligners, such as *HISAT2* [21, 22] and *STAR* [23], are suitable to map simple deletion-type recombination events. There have also been reported tools that can discover and annotate different types of recombination events found within viral genomes, including host-to-virus recombination/integration sites (*ViralFusionSeq* [24], *VERSE* [25]), copy-back and snap-back DVGs (*DI-Tector* [26], *VODKA* [27]), and intrastrain homologous recombination events (*Hexahedron* [28], *MosaicSolver* [29]). Many of these pipelines require prior knowledge/information to resolve recombination events, and they make assumptions as to the “types” of events that are found. However, due to the diverse and complex nature of viral recombination, the capture and quantification of the full range of genetic products of viral recombination require a similarly diverse and versatile computational tool.

We previously reported an algorithm for detecting and counting recombination events found in NGS analyses of viral genomes that we called “*ViReMa*” (**Vi**ral **Re**combination **Ma**pper) [30]. *ViReMa* bootstraps the *bowtie* or *bwa* short-read aligners [31, 32] to map short Illumina reads to a reference genome. For reads in which only a partial mapping is found, *ViReMa* extracts the unmapped portion of the read and remaps this segment in a new mapping iteration. If the subsequent segment maps discontinuously, then *ViReMa* reports a recombination event. Importantly, the mapping of the subsequent segment is completely independent of the previous segment, which allows *ViReMa* to identify any kind of recombination event among either strand of any reference sequences provided (virus, host, or otherwise). As a result, *ViReMa* can “agnostically” report any complex recombination event.

*ViReMa* has been independently validated by Alnaji et al. [33] and Boussier et al. [34], who provided a series of condition and parameter optimizations using simulated and experimental data to map RNA recombination events in DVGs of influenza A and B. *ViReMa* has been used and validated to study viral recombination in a range of plant viruses (polygonum ringspot virus [35], tomato yellow leaf curl virus [36], potato virus X [37]), insect viruses (Flock House virus [FHV] [38], cricket paralysis virus [39]), arboviruses virus (Zika virus [40], Chikungunya virus [41, 42]), human pathogens (influenza virus [43], Reovirus [44], Middle Eastern respiratory virus coronavirus, and SARS-CoV-2 [45, 46]), and retroviruses (HIV [7]). *ViReMa* has been employed to enumerate large-scale recombination events, such as the large recombination events that give rise to the subgenomic mRNAs synthesized during coronavirus replication [45, 46], and to characterize specimens even when the recombinant product is the predominant species, such as during the selection of defective RNAs of FHV during experimental passaging [38].

NGS technologies have advanced since the original inception of *ViReMa*, when NGS read lengths were seldom longer than 100 nts. As a result, the original pipeline accounted for only a limited number of mismatches within a mapped read segment. Here, we introduce a newer error-handling process that assigns recombination junction breakpoints once a threshold number of reference mismatches are encountered within a specific moving window, rather than simply counting the number of mismatches found in total across a mapped read segment. This is important because NGS sequencing reads are now considerably longer than when *ViReMa* was originally conceived, for example, with MiSeq reads being up to 300 bp in length. Furthermore, this allows *ViReMa* to accurately map reads from virus populations that have extensive intrahost diversity and so contain multiple minority variants relative to the reference genome.

We have also developed *ViReMa* to provide outputs in standardized bioinformatic conventions. Foremost, *ViReMa* provides alignments in SAM format [47] to allow visualization and integration with common downstream bioinformatic tools. Deletions and duplication events, as well as more complex recombination events, such as copy-back or intergenic fusions, are reported in standardized BED or BEDPE formats [48]. BED files can be loaded into genome browser tools such as *IGV* [49] or *Tablet* [50], and we recently provided an online tool “*ViReMaShiny*” to interactively generate recombination plots and figures for data visualization and interpretation [51]. We have also added a simple GUI functionality, improving accessibility as well as a Docker image packaged with necessary dependencies for cross-platform use. To illustrate the broad functionality of *ViReMa*, here we analyze multiple different model virus systems, including FHV (+ssRNA) for the detection of deletion-type recombination events, Sendai virus (−ssRNA) for the detection of copy-back DVGs, HIV (a retrovirus) for the detection of short indels and genomic duplications, and *Sulfolobus* turreted icosahedral virus (STIV) (dsDNA archaeal virus) for the detection of virus-to-host recombination junctions. Overall, *ViReMa* provides a validated and robust “1-stop” solution to simultaneously capture the full range of recombination events found within virus populations.

## Methods

### Description of the algorithm

*ViReMa* (RRID:SCR_000566) is a python3 script that bootstraps the small-read mappers: *bowtie*(RRID:SCR_005476) [52] or *bwa*(RRID:SCR_010910) [53]. Virus references are provided by the user in FASTA format (an index is automatically generated if one is absent), and the host reference must be a prebuilt *bowtie* or *bwa* index. For short reference sequences without poly(A)-tails, additional As or other artificial sequences can be added to the end of the reference genome to allow *bowtie* to map reads that would otherwise overhang the end of the virus reference genome. In the absence of this, such reads would be rejected by *bowtie* preventing *ViReMa* from finding recombination events or chimeric reads that involve the very 3′ end of the viral genome. If such pads are added, care must be taken when viewing the output data that recombination events into these “pads” are not considered to be authentic, particularly if poly(A) stretches or similar are abundant in the original dataset. The expected input for *ViReMa* is FASTQ data from short-read sequencing platforms such as Illumina. Typically, input data should be quality filtered and adaptor trimmed using, for example, *fastp*(RRID:SCR_016962) [54] prior to analysis, although this is not required. Known contaminants could also be removed prior to analysis either using *k*-mer–based filtering strategies (e.g., using *BBDuk* from the *BBMap* suite; RRID:SCR_016965) or removing reads that align to known contaminant genomes such as mycoplasma.

*ViReMa* uses *bowtie* [52] or *bwa* [53] to generate alignments of a fixed seed length starting from the leftmost position of a sequence read. After this initial mapping, *ViReMa* tracks along the rest of the read segment following the mapped seed until a disqualifying mismatch is found, as would occur at the junction of a recombination event. Mismatches are not uncommon in short reads due to errors in base calling or biological variation. Therefore, 0 to 2 mismatches are tolerated in the mapped read before a junction is inferred. Single mismatches are not allowed to occur within “X” nucleotides of the junction (the “X” value can be specified in the command line; default value is 5).

With improvements seen in NGS and the common usage of longer Illumina reads up to 300 nts in length, permitting only 2 mismatches per read became insufficient. Indeed, defining the number of allowed mismatches as a function of read length lacks biological rationale as read length is a purely technological choice, and the number of mismatches is often determined by the base-calling error rate inherent to sequencing platforms and the intrahost diversity of the sample in question. Therefore, we have introduced a new mapping procedure. The basis of mapping is the same, whereby an initial seed is mapped using *bowtie* [52] or *bwa* [53], and then trailing nucleotides are assessed for evidence of a break. However, rather than counting the total number of nucleotide mismatches to locate a breakpoint, a breakpoint is elicited when a given number of mismatches are found within a defined window. These figures are given in the command line by an “error density” parameter. The default is [1, 25], which means that up to 1 mismatch is tolerated within any 25 given nucleotide window. Two mismatches within 26 nucleotides would be tolerated. Two within 25 would invoke a recombination breakpoint at the most downstream mismatch as per the usual *ViReMa* protocol. This process has the primary advantage over the previous versions of allowing longer reads to be mapped before premature breakpoints are invoked due to simple mismatches.

### SAM output

We have substantially overhauled and updated *ViReMa* in view of making the software more user-friendly and accessible. *ViReMa* standardizes the output, returning canonical SAM files with appropriate CIGAR 1.4 scores and associated SAM tags. The purpose of this was to allow users to visually inspect their alignments using common alignment visualization software such as the Tablet viewer (RRID:SCR_000017) [50] or the Integrative Genomics Viewer (IGV) (RRID:SCR_011793) [49, 55] and allow *ViReMa* to be implemented as part of routine RNA sequencing pipelines that require SAM files as an input.

For the most part, straightforwardly mapped reads either with or without soft pads simply use the output SAM information from the original *bowtie* or *bwa* alignment. Similarly, straightforward recombination, deletion, and/or splice events are reported as canonical alignments with either “D” or “N” nucleotides in between each mapped segment. The choice of whether to specify a gap as a deletion (“D”) or a recombination/splice event (“N”) is in the command-line entry by the “*–MicroInDel*” option, with a default value of 5 nts. Insertion events shorter than the value defined by the “*–MicroInDel*” option are stored using “I” in the CIGAR string. Longer insertions are stored as soft pads (“S”) in between 2 independently reported read segments. If longer insertions are in fact the result of duplication of a segment of the underlying genome reference (as illustrated in Fig. 2), these can be reported as insertions in a single entry of the SAM file rather than 2 segments by invoking the “*–back-splicing*” option. This allows these features to more easily be visualized in NGS alignment software.

*ViReMa* is also capable of mapping complex recombination events, such as when a number of inserted nucleotides that do not map to the reference genome are present between 2 or more mapped segments of a recombination event. Awareness of these types of complex recombination events is important in the context of understanding viral recombination, as mismatched nucleotides inserted into nascently replicated RNA/DNA strands may be a trigger for nonhomologous recombination and template switching. These unmapped/unmappable nucleotides are stored as soft pads (“S”) in the output SAM file. These may occur at the end of mapped read segments or in between mapped read segments. Similarly, reads or read segments that are smaller than the fixed seed length defined in the command line of *ViReMa* and required by the aligner (e.g., *bowtie/bwa*) are not aligned and are reported either as entirely unmapped (SAM flag = 4) or as soft pads at the end of a partially mapped read.

One of the advantages of using the *ViReMa* software is the ability to map unusual recombination events, such as *trans*-splicing events, intergenic recombination, or large back-splicing or back-recombination events. Currently, there is no standardized system with which to report these types of alignments without treating each mapped segment separately in the SAM file. In *ViReMa*, we do the same, treating these types of events as chimeric reads. Individual segments are specified using the “TC:i:n” and “FC:i:n” tags, and these reads are hard-padded at the segment break junctions.

### Annotation of recombination events in original formats

All the original formats output of *ViReMa* have been retained to allow backward compatibility, as previously described [30]. Briefly, recombination junctions are output into text files with the simple format (for example): “*1102_to_1037_#_9*.” This means that there is a recombination junction with a donor site at nucleotide 1102 to an acceptor site at nucleotide 1037 in the reference genome and that 9 unique reads mapped over this junction. These junction annotations are listed underneath a header that describes the genomes that were mapped to either side of the junction (e.g., “*FHV_to_FHV*” or “*HIV_RevStrand_to_HIV_RevStrand*”).

However, we have made 2 simple additions to allow further scrutiny of these events. By invoking the “*-FuzzEntry*” option, the amount of microhomology found at the recombination junction is reported, for example: “*1102_fuzz-5_1037_#_9*.” This means that there is a recombination junction with a donor site at nucleotide 1102 to an acceptor site at nucleotide 1037 in the reference genome, that there are 5 nucleotides of microhomology at the recombination junction, and that 9 unique reads mapped over this junction. We have also added a “*-ReadNamesEntry*” option that, when invoked, appends the read name of every unique read that mapped to each reported event after the annotation. This has applications when searching for specific reads in alignment visualizers to “spot-check” and verify the authenticity of reported recombination events and may have applications in future single-cell sequencing pipelines that retain cellular barcodes and UMIs in the read names.

### Annotation of recombination events in standardized BED formats

We have updated *ViReMa* to provide recombination event annotations using standardized BED and bedgraph formats to allow visualization and integration with only bioinformatic pipelines. Virus recombination events within viral genes are expressed using canonical BED format, similar to RNA splicing events or DNA recombination events, but with a minor alteration. As of *ViReMa* version 0.25, the first 6 columns are in BED6 format, with 4 additional bespoke columns:

1. Reference name taken from FASTA file of aligned genome
2. Nucleotide coordinate of donor site
3. Nucleotide coordinate of acceptor site
4. Name of the type of event: “*Deletion*” if acceptor site is downstream of donor site, “*Duplication*” if donor site is downstream of acceptor, and “Ins:NNN” if a small insertion of “NNN” nucleotides is found or if “*–BackSplice_Limit*” is set
5. The number of mapped reads reporting this event
6. The strand of the reference genome in which the event is found (“+” or “−”)
7. The total read coverage found at the donor site (includes all reads either with or without evidence of the recombination junction at this site)
8. The total read coverage found at the acceptor site (includes all reads either with or without evidence of the recombination junction at this site)
9. The nucleotide sequence found surrounding the recombination junction of the donor site
10. The nucleotide sequence found surrounding the recombination junction of the acceptor site

The inclusion of read coverage at the recombination junctions allows for an approximation of the recombination event abundance in the dataset. However, care must be taken when performing these calculations due to the unevenness and/or bias of read coverage at different sites in a viral genome, which may result in different coverage at either recombination junction. For columns 9 and 10, the number of nucleotides reported upstream and downstream of the recombination junction is determined by the mapping seed used in the alignment (default is 25 nts), and the exact breakpoint mapped is indicated using a pipe: “|.” This provides a convenient output to assess for evidence of nucleotide bias at the sites of mapped recombination events, as well as quickly assess whether there is microhomology between recombination events, as is commonly seen, for example, in coronaviruses [45]. If the “*–MicroInDel*” option is specified in the command line, an additional BED file is produced containing the annotations only for the microindels.

**Figure 1:**
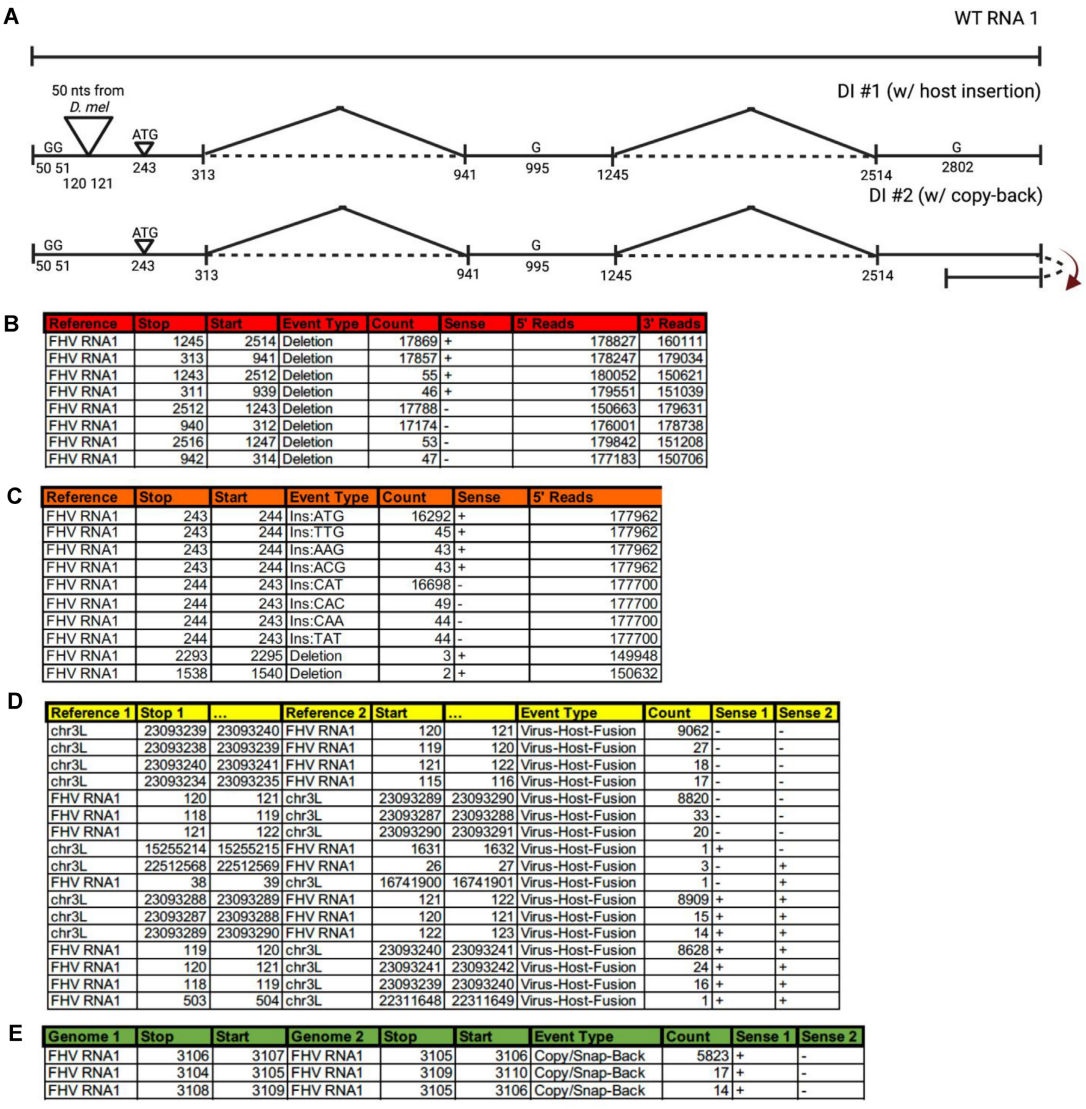
*ViReMa* analysis of simulated Flock House virus (FHV) data. (A) Defective RNA1 (D-RNA1) genomes were constructed and simulated reads were generated using “ART.” Wild-type FHV RNA1 is depicted, with schematics of hypothetical D-RNAs shown below. D-RNA1 #1 includes a 50-nt insertion from *Drosophila melanogaster* as well as 2 deletions. D-RNA1 #2 includes the same deletion events and a duplication event at the 3′ end. (B) *ViReMa* results of the simulated data are reported in the Virus_Recombination_Results.bed file. Output indicates the reference (in this case, FHV has 2 references, one for each strand of its bipartite genome), coordinates of the recombination breakpoints, the type of recombination event, the number of reads mapping to this event, the strandedness of the recombination event, and how many total reads at the 5′ and the 3′ ends of the recombination junction. (C) *ViReMa* results for simulated data found in the MicroRecombinations.bed file. These indicate the reference, the genome position when the event occurred, the type of event (Ins:XXX is an insertion of XXX nucleotides), coordinates of the recombination breakpoints, and number of reads at the 5′ and the 3′ sites of the recombination junction. (D) *ViReMa* results for simulated data found in the Virus-to-Host-Recombination.BEDPE file. This displays the reference of the first genome mapped in reads and where that stops before mapping to the second reference at the “start” nucleotide position. The event type is also noted, as well as the count of that recombination event and the strandedness of the genome of each mapped segment. (E) *ViReMa* results for simulated data found in the Virus_Fusions.BEDPE file. Similar information is depicted as in the Virus-to-Host-Recombination output. Here we show the detection of the 3′ end duplication we included in our simulated dataset.

For intergenic and/or forward-to-reverse strand recombination events, we have adopted the BEDPE format [48]. This format is illustrated in Figs. 1,3 and 4 and can be used to describe end-to-end fusions of multipartite RNA genes, intergenic recombination events, copy/snap-back DVGs (examples shown in table), and virus-to-host fusion/chimeric events.

As of *ViReMa* version 0.25, the BEDPE format contains the following information:

1. Reference name of the first mapped reads segment
2. Coordinate of the nucleotide immediately upstream of the recombination/fusion junction of the first reference (given in column 1)
3. Coordinate of the nucleotide immediately downstream of the recombination/fusion junction of the first reference (given in column 1)
4. Reference name of the second mapped reads segment
5. Coordinate of the nucleotide immediately upstream of the recombination/fusion junction of the second reference (given in column 4)
6. Coordinate of the nucleotide immediately downstream of the recombination/fusion junction of the second reference (given in column 4).
7. Name of the type of event: “*Copy/Snap-Back*” if read segments map to different strands of same viral genome, “*Intergenic-Fusion*” if read segments map to different viral genes and or viral genomes, “*Virus-Host-Fusion*” if 1 read segment maps to a viral gene and 1 read segment maps to the host genome, and “*Gene-Fusion*” if read segments map to different host genes/chromosomes
8. The number of mapped reads reporting this event
9. The strand of the reference genome given in column 1 (“+” or “−”)
10. The strand of the reference genome given in column 4 (“+” or “−”)

A “*Virus-Host-Fusion*” event may describe a direct fusion of viral and host nucleic acids (such as described below for STIV) or the integration of the viral nucleic acid into the host genome, such as would be expected for an HIV provirus. Differentiating between these 2 scenarios depends upon the experimental setup prior to *ViReMa* (e.g., whether the input material sequenced derived from the host nucleus or virions) and upon downstream interpretation of the results reported by *ViReMa* (e.g., whether the recombination/fusion breakpoints in the viral genome are located at the expected loci, such as the Long Terminal Repeats (LTRs) of HIV).

### Additional features and optimizations

By profiling the rate/usage/time of each operation in the script, we determined that ∼50% of the runtime can be attributed to each iterative *bowtie* alignment. As a result, datasets that have a large number of unmappable reads or reads that would map to a reference that is not provided will incur several rounds of futile iterative alignments. We have added a feature invoked using “–*MaxIters*” that allows users to limit the number of iterations that are attempted. This prevents long runtimes associated with unmappable reads being repeatedly tested. This option may be important in scenarios where users do not have access to a complete assembled genome for the host and/or when the host material is the predominant species in the input RNA/DNA that was originally sequenced.

As unmapped reads are stored in temporary memory, there is a natural limit to the number of reads that can be aligned in one instance of *ViReMa*. As a result, very large datasets may get “stuck” in the initial phases of the program's initialization. We have added a feature invoked using “–*Chunk*” to allow users to analyze large datasets in “chunks,” whereby a user-defined number of reads are mapped in any given time. We set a default “chunk” size of 1 million reads. This number was chosen as it typically corresponds to a requirement for approximately 1 to 2 Gb of RAM, which is easily accessible for most regular workstations and does not saturate the 2-Gb limit imposed by *pypy*.

### Simulated data

The ART suite of tools (RRID:SCR_006538) for the simulation of NGS reads was used to generate datasets of reads of synthetic recombination events and defective RNAs (D-RNAs) of FHV [56]. Parameters used were 37,000× coverage over each of the 2 simulated D-RNAs and 300,000× over the wild-type virus. We generated individual FASTQ files for each synthetic FASTA reference file, and reads were concatenated from multiple FASTQ files in ratios stipulated in the main text to generate single FASTQ files used in the *ViReMa* analysis. An error profile reflecting the HiSeq 2500 was imposed for reads 100 bases in length, yielding ∼10 million reads.

### Data alignments and visualization

All data were aligned to the virus genomes either with or without the host genome in parallel using *ViReMa* version 0.25 (RRID:SCR_000566) [30] using parameters and command lines described in the main text for each virus sample. Output SAM files were transformed and sorted into BAM files using *samtools*(RRID:SCR_002105) [47], and coverage plots were obtained using *bedtools*(RRID:SCR_006646) [48]. Read alignments were visualized using *Tablet* (RRID:SCR_000017) [50].

## Results

### FHV recombination in simulated data

We generated 2 artificial D-RNAs based upon recombination events previously seen in FHV, as well as imaginary events designed to challenge and test our platform. These templates are illustrated in Fig. 1. The first D-RNA contained 2 deletion events, a simple 3-nt insertion, multiple point mutations, and an insertion of a 50-nt fragment of host mRNA (chr3L:23,086,340–23,086,389 from *dm6*) at nt120 of the viral genome. The second D-RNA, instead of containing this host insertion event, contained a fusion-type recombination event (akin to a copy-back RNA), whereby the 3′ end of the viral genome is fused to the reverse sense of the 3′-most 41 nts of the genome. Using these input reference sequences, we generated simulated reads using “ART” tools [57] requiring approximately 37,000× coverage over each simulated D-RNA (74,000× total for both D-RNAs) and 300,000× over the wild-type virus. An error profile reflecting the HiSeq 2500 was imposed, yielding 10,935,310 reads.

We ran the *ViReMa* pipelines using standard parameters: (–X 3 –Defuzz 0 –Host_Seed 25 -BED –MicroInDel_Length 5) mapping to both a padded FHV genome and *Drosophila melanogaster* (*dm6*) genome. The resulting BED files produced are shown in Fig. 1B, C and Supplementary Data S1. As can be seen, each of the simulated recombination events is successfully detected with no erroneous mappings. The correct deletion recombination events at 313^941 and 1245^2514 are found, as is a small ATG insertion between nts 243 and 244. The expected host insertion (virus–host fusion type) event was successfully detected, as reported using the BEDPE output (Fig. 1D). Both virus-to-host junctions were found corresponding to both ends of the insertion and with reads mapping in both the negative and positive sense orientation. Finally, the hypothetical positive to negative sense recombination event at the 3′ end of the viral genome (illustrated in Fig. 1A) was also captured, as reported using the BEDPE output (Fig. 1E).

As the coverage of simulated reads over the defective genomes is approximately 37,000× for each D-RNA, we should therefore expect to find approximately 74,000 or 37,000 reads that map over either the shared or unique (respectively) synthetic recombination junctions illustrated in Fig. 1A. However, recombination junctions cannot be found in the extremities of individual sequence reads, as there are not enough bases available to unambiguously map either side of a recombination junction. With a seed length of 25 nt and with 100-nt-long reads, there are only 49 potential “cutting sites” [30] per read across which recombination junctions can be detected in a single read. Therefore, we can maximally only expect to find 36,260 or 18,130 reads that map over either the shared or unique (respectively) synthetic recombination junctions.

As seen in Fig. 1C, *ViReMa* found 35,031 and 35,657 reads (combination of both positive and negative orientation) mapping to the deletion recombination events at 313^941 and 1245^2514, respectively, yielding ∼97% mapping efficiency. The imperfect sensitivity is due to the presence of single-nucleotide mismatches that are found near recombination junctions. While these reads can be found in the output SAM file, *ViReMa* does not annotate these reads as containing recombination events if mismatches are found near the putative recombination junction at a distance defined by the optional –X parameter (set to 3 above). This value can be adjusted to increase sensitivity but at the cost of introducing false-positive events [43]. As the host insertion event was only present in one of the synthetic D-RNAs, we would expect approximately 18,130 reads to map to either side of the insertion event. As seen in Fig. 1D, we detected 17,729 and 17,690 (combination of both positive and negative orientation) mapping to each end of the host insertion event, again yielding ∼97% mapping efficiency.

As the duplicated, negative-sense portion of this event was only 41 nts in length, this restricts the number of the 100-nt-long reads that can map to this region as *bowtie* does not allow reads to overhand the ends of a reference genome. Therefore, with a seed length of 25 nts, there are only 16 [15–17] possible cutting sites remaining in any read across which this junction can be detected. From an expected coverage of 18,500×, we would therefore expect approximately 5,920 reads. ViReMa found the copy-back-like recombination event at the 3′ end of the viral genome with 5,823 reads (Fig. 1E), therefore yielding an efficiency of ∼98%.

### Duplications in the GAG region of the HIV genome

**Figure 2:**
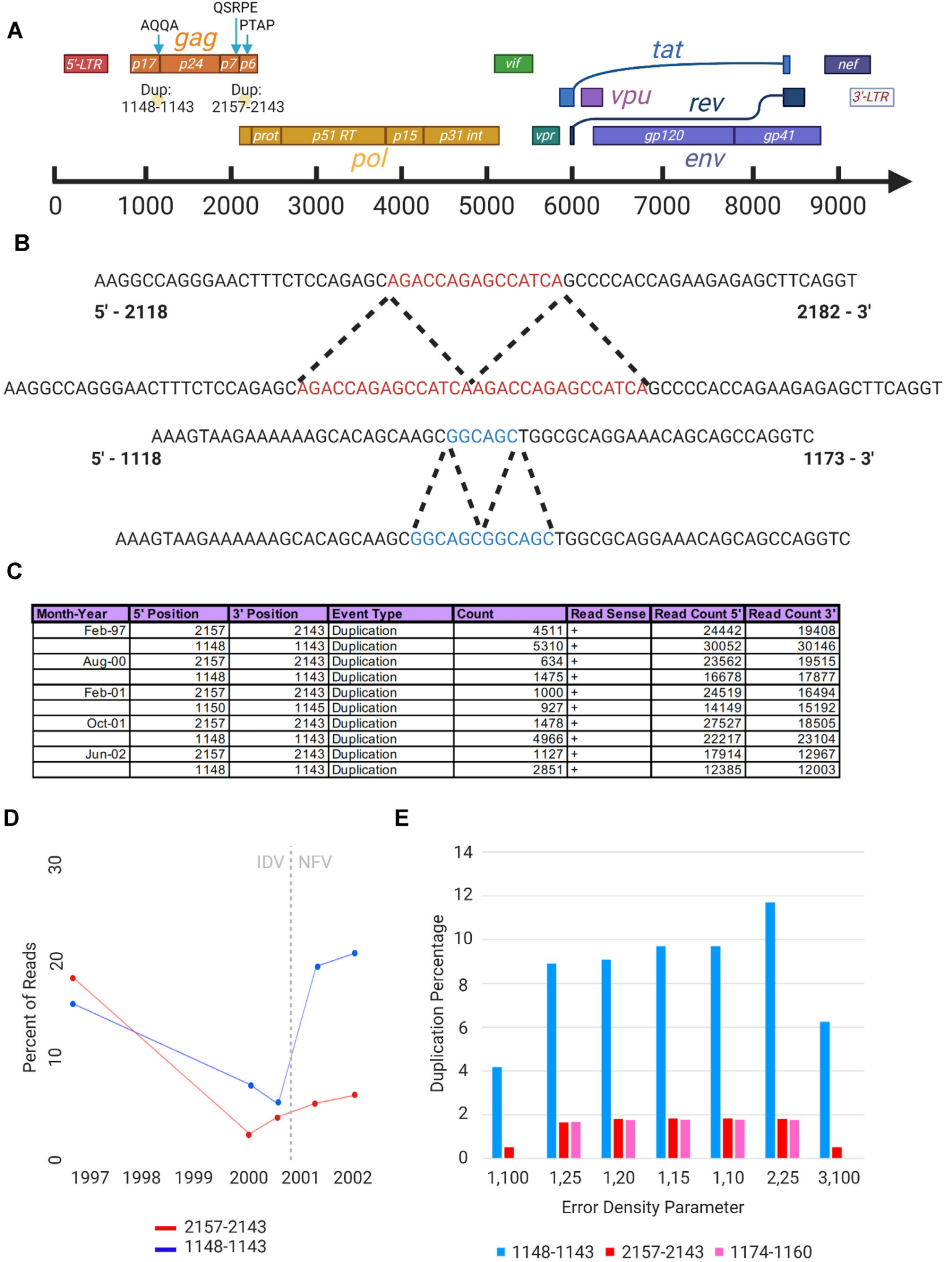
Duplications in *gag* in HIV patient data. (A) Schematic of the HIV genome with the identified duplication events noted at nucleotide positions 1148–1143 and 2157–2143. (B) Depiction of these duplication events at the sequence level. (C) *ViReMa* output in the Virus_Recombination_Results.bed with all time points compiled and focused on the primary duplication events. (D) Plot of the percentage of mapped reads at these nucleotide positions that identify the noted duplication events over time. Note that the patient's antiretroviral treatment was changed after the third time point from indinavir (IDV) to nelfinavir (NFV). (E) Bar chart depicting the number of duplication events using different “error density” parameters in the command line. The parameters are entered as “x,y,” where x is the number of allowed mismatches in y nucleotides. We include the 2 abovementioned events and an additional duplication event at position 1174.

In a previous study, we reported the analysis of covariation of amino acid and nucleotide variants within a large cohort of HIV-1 and antiretroviral therapy patients who were part of the US Military HIV Natural History Longitudinal Study [58, 59]. These datasets were obtained by sequencing complementary DNA (cDNA) amplicons derived from template-specific reverse transcription PCR of the gag-pol genes. We used *ViReMa* to detect insertions, deletions, or recombination events in HIV obtained from patient blood samples during antiretroviral therapy. To begin, processed reads were mapped to the reference HIV-1 genome (Fig. 2A, B) (NL4-3) using *ViReMa* (BED files are provided in Supplementary Data S2). We invoked the *–back-splicing* option to report short insertions and duplications in the HIV genome, as described in the Methods. This revealed a large number of recombination events occurring close to the protease cleavage sites between p17 (matrix) and p24 (capsid) proteins, as well as in the PTAP region of the p6 protein (Fig. 2A). These events are largely characterized by in-frame duplications ranging from 3 to 24 nts in length. The most common HIV sequence alterations resulting from resistance to inhibitor treatment in patients group into 3 HIV genomic regions and are as follows (Fig. 2A): (i) small duplications at the “AQQA” motif found 13 amino acids upstream of the p17/24 protease cleavage site, (ii) “QSRPE” duplications 2 amino acids downstream of the p7 (nucleocapsid)/p6 (p1p6) protease cleavage site, and (iii) “PTAP” duplications 10 amino acids downstream of the p7/p6 protease cleavage site. Importantly, these duplications have been previously observed and have been characterized as a response to antiviral drug treatment [59–62]. There is some variation in the exact nature of these duplications, varying in length from 3 to 24 nts, sometimes containing inexact duplications that introduce variant amino acids at the duplication site. Nonetheless, the same or closely similar events were seen in multiple different patient samples, indicating that these duplications were a response to a common evolutionary selection pressure. In Fig. 2A and B, we illustrate where AQQA and PTAP duplications are occurring in the context of the HIV genome and what these duplications are at the nucleotide level for 2 examples: a “PTAP” duplication with coordinates 2157^2143 and an “AA” duplication with coordinates 1148^1143. Note that in the third time point (February 2001), the 1148^1143 is replaced with 1150^1145, shown in the table in Fig. 2C. The gray dotted line in panel Fig. 2D illustrates when the patient was switched from indinavir (IDV) to another antiretroviral—nelfinavir (NFV). After switching from IDV to NFV, the patient showed a substantial increase in the abundance of the “AA” duplication and only a small increase in the PTAP duplication.

We recently reported that SNVs elsewhere in the viral genome are evolutionarily corelated with these duplications [59], some of which were found in close proximity to the duplication site (e.g., SNV D121A and duplication event 1148^1143). Given the high intrahost diversity of the HIV population in these samples, careful consideration must be taken about the number of reference sequence mismatches that are tolerated during the *ViReMa* alignment process. If too few mismatches are allowed in each segment, then recombination events found near to SNVs will not be reported if the gap between the SNV and the recombination event is smaller than that selected mapping seed. We therefore used these data to investigate how changing the mismatch handling parameters affected the read alignment and recombination detection of *ViReMa*.

We mapped the February 2001 data using *ViReMa* with a range of error-handling parameters listed in Fig. 2E. Using the “error density” function allowing no more than 2 mismatches within a 25-nt window yielded a small increase of ∼10% in the number of mapped reads as well as a greater proportion of reported recombination events (∼10% increase). The more permissive settings (1,10 and 1,15) increased the number of mapped reads by a further ∼3% but did not change the proportion of these that reported recombination events. The largest deviations in duplication events per reads at these particular nucleotide positions were found when using the least permissive parameters of 3,100 and 1,100, which, as expected, reduced both the number of reads mapped and the overall proportion of these reporting recombination events. This illustrates that a more permissive handling of mismatches surrounding putative recombination events can have an impact on how frequently they are reported. Altogether, these HIV patient datasets illustrate the ability of *ViReMa* to identify duplication events and how altering the permissibility of *ViReMa* by changing the error density parameter allows the detection of drug-resistant duplications that would otherwise have been missed due to their correlation to other drug-resistant SNVs.

### Copy-back DVGs are detected in the negative-sense RNA virus, Sendai virus

Copy-back or snap-back RNAs are types of DVGs commonly found in negative-sense RNA viruses, including measles, mumps, Nipah virus, and Sendai (SeV) virus [12]. They emerge during the synthesis of the viral genome (negative sense) when using the antigenome (positive sense) as a template through a presumed mechanism in which the viral polymerase stalls and detaches from the template strand and begins copying the nascent strand, creating a hairpin loop with complementary sequences in the strand at both ends. The existence of copy-bask DVGs is well characterized for SeV (a paramyxovirus closely related to human parainfluenza viruses 1 and 3, also called murine parainfluenza virus 1), where sequencing found defective interfering genomes that consisted of the 5′ end of the genomic (negative-sense) strand and the 3′ end of the positive-sense or coding strand [63]. Since their original description, these recombination events have been shown to be strongly immunostimulatory and to promote persistent viral infections by preventing apoptosis in DVG-enriched cells versus highly infected cells with full-length virus [64].

**Figure 3:**
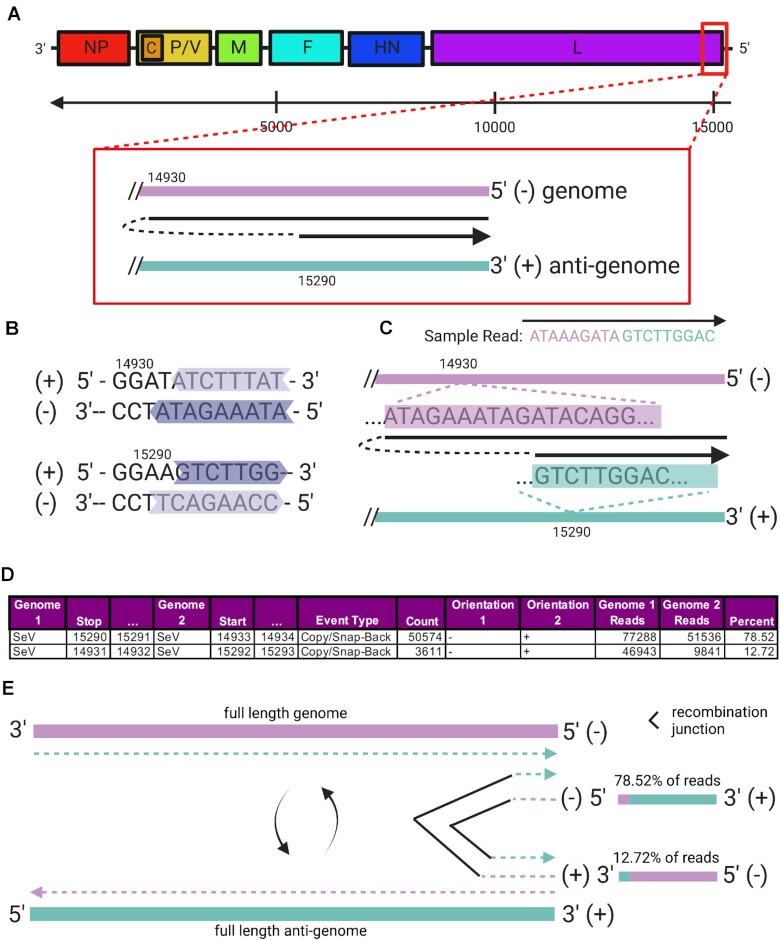
Detection of copy-back RNAs in Sendai virus (SeV) using *ViReMa*. (A) Map of the negative-sense (packaged) SeV genome with a box around the 5′ end where copy-back DVGs occur. Expanded in the box is a model for how copy-backs are made by template switching of the viral polymerase. (B) Sequences in the positive- and negative-sense strands of SeV where template switching produces copy-back RNAs. Here the arrow of each color indicates direction of the polymerase and the color indicates a particular copy-back (e.g., light purple and purple are separate copy-backs). (C) Depiction of a sample read identifying a copy-back and how that aligns to the 5′ end of the negative- sense strand and 3′ end of the positive-sense strand. (D) Table showing the number of copy-back events in the data set. The “Percent” column is calculated from the count of reads with the recombination event (from Virus_Fusions.BEDPE) compared to the total number of reads at those nucleotide positions (calculated using *bedtools*). (E) Our results indicate the presence of a secondary copy-back genome. While the primary copy-back is produced by a template-switching event from a negative-sense (genome) to a positive-sense (antigenome) template, the secondary copy-back is produced by replication using the primary copy-back as a template. The secondary copy-back accounts for 78% of reads at these genome positions (15290 [−] and 14933 [+]).

As part of a previous study investigating the roles of copy-back DVGs in SeV pathogenesis [64], total RNA was purified from DVG-enriched infected cells in culture and used to synthesize cDNA libraries using an Illumina (San Diego, CA, USA) TruSeq Stranded Total RNA LT kit with Ribo-Zero Gold. cDNA libraries were sequenced on an Illumina NextSeq 550, obtaining 21 to 53 million 75-bp single-end reads. Here, we focused on the SeV sample experimentally determined to contain a high abundance of 1 species of copy-back DVG. We mapped these reads using *ViReMa* to both the SeV genome (AB855654.1) and the human genome (hg19) to confirm the presence of known copy-back DVGs (Fig. 3). *ViReMa* detected 21 unique potential copy-back DVGs in this dataset (Supplementary Data S3), but only 4 of these were detected with more than 1 mapped read. The 2 most common events were negative- to positive-sense fusions of the SeV genome at coordinates 15291^(−)^^14933^(+)^ and 14932^()^^15292^(+)^, as illustrated in Fig. 3B and C, with 50,574 and 3,611 reads, respectively. This major copy-back DVG was also identified using *VODKA*[27]. These read counts are in far excess of the number of reads found for other types of RNA recombination events, such as a deletion-type event, with the most abundance recombination event having only 29 reads (Supplementary Data S3).

The data in the table in Fig. 3D are derived from the *ViReMa* output file “*Virus_Fusions.BEDPE*.” The “Reads” columns describe the number of reads at each particular nucleotide position of SeV, and the “Percent” column was populated by calculating the number of recombination event counts over the average of the 3′ and 5′ read counts. Although reported separately, these are the same copy-back DVGs but reflect reads mapping to both the sense and the antisense version of this DVG. Interestingly, although 14932^()^^15292^(+)^ is the junction that would be expected in the original copy-back DVG generated during synthesis from the positive-sense antigenome, the reverse-complementary 15291^()^^14933^(+)^ events is approximately 6.5-fold more abundant. As the cDNA synthesis strategy used retains the original strandedness of the input RNA, these data therefore suggest that multiple antisense DVGs are generated from the original DVG. This example demonstrates the ability of *ViReMa* to faithfully identify copy-back recombination events and use their relative abundance to reveal that DVGs are actively replicated into anti-DVGs.

### STIV captures fragments of the host genome

**Figure 4:**
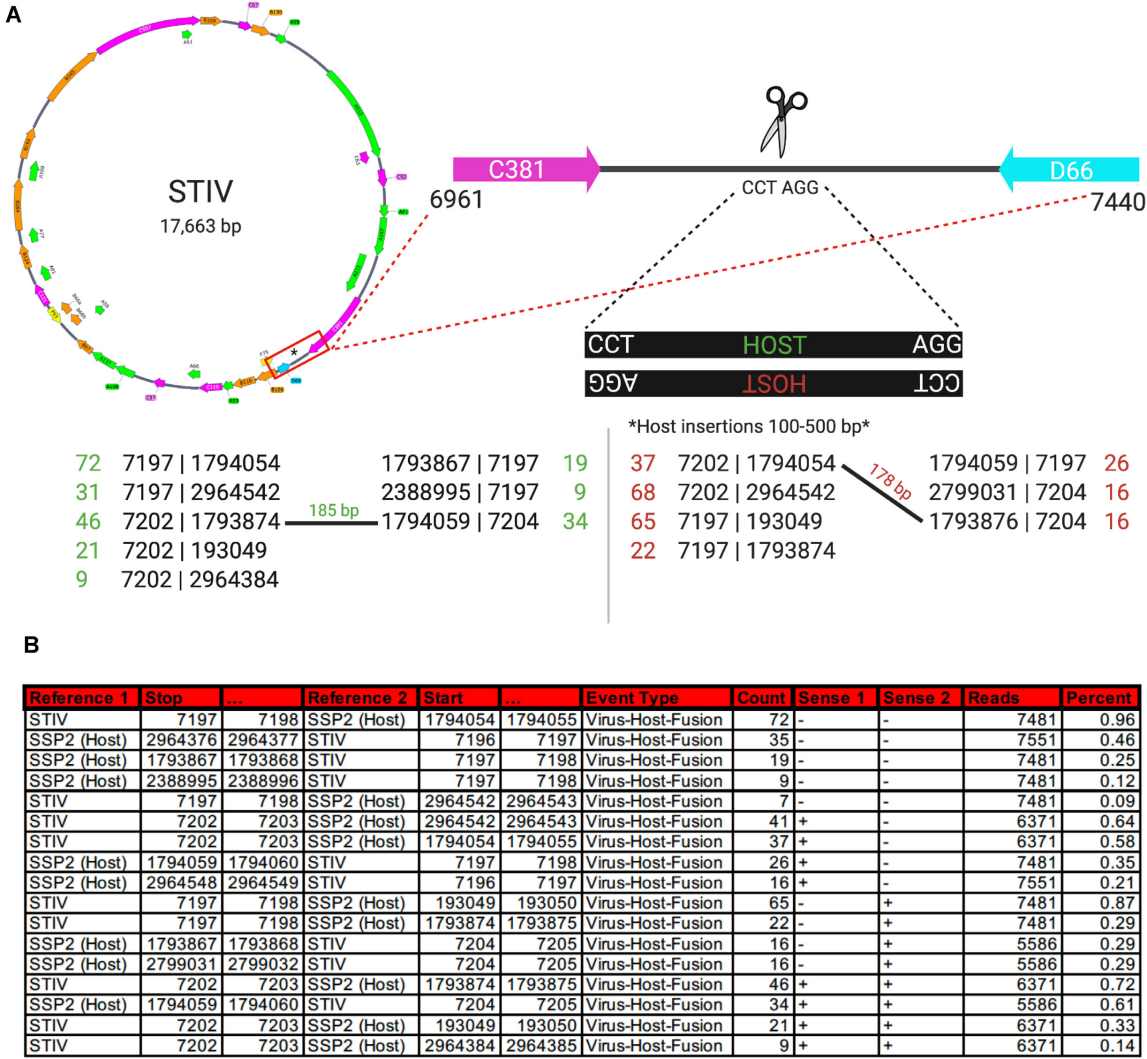
Host-to-virus recombination in *Sulfolobus* turreted icosahedral virus (STIV) as detected by *ViReMa*. (A) Map of the circular STIV genome and a zoomed-in region between genes C381 and D66 with a CCTAGG motif where host-to-virus recombination occurs. We list the recombination events found in the dataset at that site. Here, upside-down “HOST” in red is from the opposite strand of STIV, and therefore recombination occurs at the same site and the motif is in reverse complement. (B) *ViReMa* output from the Virus-to-Host_Recombinations.BEDPE. Where “Reference 1” is the first genome mapped to a read up until position “Stop,” “Reference 2” is the second genome mapped from “Start,” and “Sense 1”/“Sense 2” refers to the sense of the respective reference genome. The “Count” is the number of reads containing the fusion event, and “Reads” is the total number of reads mapped to the virus at that position (calculated using *bedtools*). “Percent” was calculated by comparing the “Count” to “Reads” at that position.

STIV infects the thermophile *Sulfolobus solfataricus* (SSP2). These archaea, and the viruses that infect them, are found in hot springs and have evolved to withstand their harsh environments. Virus particles are 74 nm in diameter, contain an inner membrane within its icosahedral capsid, and enclose a 17.7-kb double-stranded circular DNA genome [65, 66]. To characterize rates of recombination in a DNA virus, we obtained genomic DNA from purified particles of STIV expressed in culture [67]. Genomic viral DNA was prepared for NGS using ClickSeq [39] and sequenced on an Illumina HiSeq 1000 obtaining ∼36 million single-end 150-bp reads. We used *ViReMa* to map these reads to the STIV genome (NC_005892.1) and the host genome (*Sulfolobus*: AE006641.1) in parallel (BED files are provided in Supplementary Data S4). Given the large size of the dataset, the default “–Chunk” feature would have processed the reads in packets of 1 million reads at a time. This would have prevented a large number of unmapped or unmappable reads consuming the workstation's RAM space. As expected, the majority of the NGS reads mapped directly to the virus genome (Table 1). Interestingly, however, we also observed a large number of “virus-to-host” recombination events. Such recombination events are illustrated in Fig. 4A, and the output information reported in the BEDPE file is shown in Fig. 4B. The column labeled “Reads” describes the number of reads at that nucleotide position of STIV (obtained using *bedtools*), and the final column was added to highlight the percentage of viral reads at that position containing that particular recombination event.

**Table 1:** STIV mapping statistics

Total reads	36,298,490
STIV	35,831,428
SSP2	8,598
Unmapped	458,464
Virus recombination events	38,150
Host recombination events	35
Virus-to-host recombination	1,770

The recombination junction breakpoints in the viral genome were strongly enriched at nucleotide position 7200 of the STIV genome, which is in a noncoding region between the genes encoding viral proteins C381 and D66 (Fig. 4A). The recombination junction breakpoints were found at a few different loci within the host genome. Reads mapping over recombination junctions are found in both positive- and negative-sense orientations. Furthermore, many of the host recombination junctions occur very close to one another (pairs of junctions are observed within 100–500 nts of each other). These observations are all consistent with the insertion of small (100–500 nts) portions of host genome into the viral genome in both positive and negative orientations (illustrated in Fig. 4A). Interestingly, both the viral loci for these recombination junctions and all the junction sites of the inserted host segments occur at “CCTAGG” motifs. The palindromic nature of this motif is reminiscent of mechanisms employed by bacteriophages that utilize recombinases to cut and transpose the phage genome into the host genome at short complementary palindromic sites to form a lysogenic prophage. STIV has previously been shown to undergo homologous recombination with various species of *Sulfolobus* at sites with this motif [68].

We posit a mechanism akin to classical bacterial transduction, whereby STIV integrates its own genome into the host genome at “CCTAGG” sites using a viral recombinase. Upon reactivation, the CCTAGG sites at either end of the viral prophage are recut to release the viral prophage. However, if there is an additional CCTAGG site in the host genome that is close to the prophage integration site, then a small additional segment of host DNA is inadvertently introduced into the viral genome as a small insertion. As the Illumina reads are too short (150 nts) to completely map across these small host DNA insertions and into the flanking viral genome on both sides of the insertion, it is also possible that these recombination events correspond to the virus-to-host junctions of proviral integration sites into the host genome. However, the input DNA was obtained from purified virus particles expressed in culture. These data therefore demonstrate the ability of *ViReMa* to identify virus-to-host recombination events and reveal a putative capture of host DNA into a viral vector as a consequence of proviral integration.

## Discussion

*ViReMa* has provided a versatile tool for the detection and enumeration of recombination events in a broad array of virus families. *ViReMa* has been independently validated [33, 34], demonstrating robust sensitivity and accuracy. To keep up with continuous improvements in NGS platforms (such as longer read lengths) and to accurately capture recombination events found within complex and diverse populations of virus genomes, we present here a renovated handling of mismatches in aligned read segments. We also provide standardized outputs (SAM, BED, and BEDPE files) for both the read alignment and discovered recombination junctions that allow integration of the outputs of *ViReMa* with other sequence visualization software and bioinformatic packages. Using a series of “case studies,” we demonstrate how *ViReMa* can be employed to capture different recombination events. These include deletions in FHV, short duplications in the HIV genome and copy-back RNAs in SeV, and virus-to-host fusion events in STIV.

*ViReMa* strictly requires a user-defined number of nucleotides to be mapped either side of a putative recombination junction, providing confidence in the identity of the recombination event reported, though at the expense of sensitivity. For situations where the predominant form of recombination event is a deletion, canonical pipelines for splice detection such as HISAT2 [21] and STAR [23] would provide a quicker and more sensitive route to detect these events as they do not rely on multiple iterations of *bowtie* alignment and use a dynamic seed-based heuristic. Additionally, where recombination/deletion events are known *a priori*, annotated junction events can be utilized in the index building steps of HISAT2 [21] and *STAR* [23]. In these cases, however, recombination acceptor sites must be located downstream of recombination donor sites within the same single reference sequence, as would be the case in canonical eukaryotic splicing events. Additionally, these aligners may be overly permissive in requiring mapped nucleotides on either side of a recombination junction, resulting in the reporting of artifactual events. For eukaryotic splicing, this permissibility is acceptable as splice events are restricted to a small number of possible sites. However, this is not the case for viral recombination, where junctions are often diverse and unpredictable, as previously noted for RNA viruses such as FHV [38]. To reflect this, *ViReMa* enforces strict parameters for read mapping either side of a junction for all identified recombination events.

*ViReMa* does not make any assumptions with regards to expected read coverage or coverage bias over a reference sequence when making an alignment as all single reads are mapped independently from one another. Rather, the raw read counts for unique recombination junctions are provided in a simple manner, and the read coverage at the positions is provided in the BED files. Recombination “frequencies” can be calculated by comparing the number of reads mapping to a recombination event to the number of reads mapping to the wild-type sequence, as utilized above and in our previous analyses of estimating RNA recombination rates in coronaviruses [45]. However, in very low-coverage datasets and/or samples in which target-capture probes and PCR biases result in uneven genome coverage, it is possible that read counts for specific recombination junctions might be artificially inflated. It is therefore important to carefully consider the quality and depth of the mapped read data across the viral genome when making statements about recombination “frequency” or rates, for example, by visualizing the output SAM files in alignment visualization tools such as *Tablet* [50].

*ViReMa* requires the user(s) to provide at least 1 reference genome template to which read alignments will be attempted. Multiple virus genomes or genomic segments may be provided in a single FASTA file, or additional sequences may be provided in the “host” index. However, there may be situations in which 1 specific reference is not available and/or the underlying diversity in the viral genome has not been previously characterized. Without a reference genome, *ViReMa* would be unable to map any reads or recombination events. In these cases, a *de novo* assembly step should be employed to provide the fullest consensus sequence prior to running *ViReMa*, for example, using the ASPIRE pipeline [69]. Similarly, SNVs in the consensus genome should be corrected as best as is possible (e.g., using *Pilon* [70] or*V-Pipe* [71]). While *ViReMa* will still be able to map reads over SNVs and other small indels, these will be counted by *ViReMa* as a “mismatch” during the alignment phase of the algorithm. If this SNV causes *ViReMa* to exceed the number of mismatches permitted by the user-defined error settings (e.g., such as the –Error_Density feature), *ViReMa* will initiate a search for a possible recombination event. In cases where considerable genomic diversity is present or numerous minority variants and/or SNVs are expected, careful consideration must be paid to the error density settings, as described for the HIV samples analyzed above.

Recently, due to the increased interest in copy-back and snap-back DVGs in negative-strand RNA viruses such as respiratory syncytial virus [72], a number of platforms have been proposed to specifically map these species, including *DI-Tector*[26] and *VODKA* [27]. Early versions of *DI-Tector* [26] and *VODKA* [27] search for negative-strand to positive-strand fusion events that are found in the copy-back and snap-back DVGs. The VODKA algorithm will generate numerous reference sequences based upon putative copy/snap-back viral genome rearrangements and align short reads to a “pseudo-library.” This requires prior information to focus the pseudo-reference library to regions suspected or known to form DVGs. Without this knowledge, the pseudo-reference library can become exponentially large with increased genome size. However, by providing a range of putative sequences with predefined recombination junctions, this constitutes a more sensitive approach as unambiguous mappings can be found across these junctions’ sites while requiring a smaller seed region. Further, search-seed lengths and tolerance to nonreference variations can be optimized for this application. Therefore, similar to finding known deletion/splice-like events using *HISAT2/STAR* aligners, in scenarios where information about what types of putative the copy/snap-back DVGs is already known, VODKA provides a more sensitive readout. In contrast, when no prior information is available, *ViReMa* provides an “agnostic” approach but at the expense of sensitivity.

We have utilized *ViReMa* here to uncover evidence of duplications and insertions in clinical HIV isolates in response to antiretroviral therapy. In principle, the junctions of larger duplications and/or insertions of nucleic acids into viral genomes can be discovered by *ViReMa* in the same manner and can provide direct evidence of such duplications and details of the junction breakpoints. However, large duplications that exceed the length of short sequence reads or repetitive regions of some larger viruses that contain diverse gene copies, such as in orthopoxviruses, would require additional experimental data to validate and reconstruct these complex species. For example, long-read nanopore data have the capacity to map across complex and variable repetitive elements in vaccinia virus, each containing unique point mutations [73].

Currently, *ViReMa* has been validated and tested solely on high-accuracy short-read NGS data. With continued improvements to long-read sequencing platforms in terms of data yield, sequence length, and accuracy (such as the Oxford Nanopore Technologies R10.4 nanopore), future applications of *ViReMa* may include the discovery of recombination or other types of fusion events in nucleic acids not restricted to viral species. Overall, the broad utility of *ViReMa* derives from its strategy whereby read segments are mapped entirely independently from other read segments. This versatility places it as a useful all-in-one tool, particularly when *a priori* knowledge is lacking, for the discovery, mapping, and annotations of viral recombination events.

## Software Availability

Project name: ViReMa

Project home page: https://sourceforge.net/projects/virema/

Programming language: Python

License: MIT

RRID:  *SCR_000 566*

All python scripts, associated test data, and readme files as well as updates can be found open source at https://sourceforge.net/projects/virema/ and can be either downloaded manually at this page or by using wget "https://sourceforge.net/projects/virema/files/ViReMa_0.25/ViReMa_0.25.zip.”

Instructions on how to analyze the small-example datasets and to validate correct installation are provided in the associated README.txt file. A docker image and associated documentation is provided at https://github.com/Routh-Lab/ViReMaDocker.

## Supplementary Material

giad009_GIGA-D-22-00132_Original_Submission

giad009_GIGA-D-22-00132_Revision_1

giad009_Response_to_Reviewer_Comments_Original_Submission

giad009_Reviewer_1_Report_Original_Submission Diogo Pratas -- 6/7/2022 Reviewed

giad009_Reviewer_2_Report_Original_Submission Fadi G Alnaji -- 7/1/2022 Reviewed

giad009_Reviewer_2_Report_Revision_1 Fadi G Alnaji -- 1/17/2023 Reviewed

## Data Availability

Supplementary data associated with this article, including BED files and simulated Flock House virus NGS data, are found at Sourceforge [74]. An archival copy of the code and supporting data is also available via the *GigaScience* database GigaDB [75]. Raw data from HIV datasets are publicly available in NCBI SRA under the accession codes SRR15732332, SRR15732333, SRR15732334, and SRR15732336. Sendai virus data are publicly available in NCBI GEO with accession GSM2543124. *Sulfolobus* turreted icosahedral virus data are publicly available in NCBI SRA with bioproject number PRJNA819882.

## Additional Files

**Supplementary Data S1**. Output files in BED format from *ViReMa* analysis of simulated flock house virus (FHV) data.

**Supplementary Data S2**. Output files in BED format from *ViReMa* analysis of clinical specimens of HIV across 5 time points.

**Supplementary Data S3**. Output files in BED format from *ViReMa* analysis of purified Sendai virus (SeV).

**Supplementary Data S4**. Output files in BED format from *ViReMa* analysis of *Sulfolobus* turreted icosahedral virus (STIV).

All supplementary data can be found online at https://sourceforge.net/projects/virema/files/SupplementaryData_Sotcheff-et-al/

## Abbreviations

AA: amino acid; bp: base pair; cDNA: complementary DNA; D-RNA: defective RNA; DVG: defective viral genome; FHV: Flock House virus; IDV: indinavir; IGV: Integrative Genomics Viewer; mRNA: messenger RNA; NGS: next-generation sequencing; NFV: nelfinavir; nt: nucleotide; SARS-CoV-2: severe acute respiratory virus coronavirus 2; SeV: Sendai virus; SNV: single-nucleotide variant; STIV: *Sulfolobus* turreted icosahedral virus; *ViReMa*: Viral Recombination Mapper.
