# Supplementary material for: ViReMa: a virus recombination mapper of next-generation sequencing data characterizes diverse recombinant viral nucleic acids
Source: Gigascience. 2023 Mar 20;12:giad009. doi: 10.1093/gigascience/giad009 (PMC10025937; doi:10.1093/gigascience/giad009)
Supplement: giad009_Response_to_Reviewer_Comments_Original_Submission [file giad009_response_to_reviewer_comments_original_submission.pdf]

Author's Response To Reviewer Comments

Close

(The text box provided does not allow for formatting of reviewer vs author responses, and so may be a little unclear. please s

Reviewer #1: This article describes a pipeline (coded in Python) to detect and analyze recombination events of viral genomes
that typically are unique architectural designs and important alternative ways to the area, including several results.

We would like to thank the reviewer for their time and effort reviewing our manuscript and appreciate their constructive comm

However, many points require attention, namely:

1) This pipeline expects exactly a specific virus. Hence, it uses a specific reference. However, this reference might not be the
more significant variations (without de-novo support), it is exclusively for targeted support. Therefore, the article could be mo

2) The article states that the improvement is also inspired with the read length increase that NGS is bringing. Also, the report

Thank you for these points. These are important considerations, and we have added the following text to the discussion:

"ViReMa requires the user(s) to provide at least one reference genome template to which read alignments will be attempted.
underlying diversity in the viral genome has not been previously characterized. Without a reference genome, ViReMa would b
(70). Similarly, single-nucleotide variants (SNVs) in the consensus genome should be corrected as best as is possible (e.g. us
the number of mismatches permitted by the user-defined error settings (e.g. such as the --Error_Density feature), ViReMa w
described for the HIV samples analyzed above."
However, it does not matter what the 'size' or 'predominance' of a recombination event is, as reads and read 'segments' are
"ViReMa has been employed to enumerate large-scale recombination events, such as the large recombination events that giv
Flock House virus during experimental passaging."

3) About the use of artificial poly-(A)tales to allow the mapper to align the reads, what happens when the read size is smaller
are very high-depth coverages? Please, comment on this matter.

Reads or read segments that are smaller than the k-mer size defined in the command-line of ViReMa and thus in the seed-ba
successfully aligned, this segment is appended as a soft-pad to the SAM entry. To clarify, we have added the following text in

"Similarly, reads or read segments that are smaller than the fixed seed length defined in the command-line of ViReMa and re

With regards to artificial poly(A) tails; this can indeed result in issues if poly(A) stretches are found in NGS reads as these mi
final BED file. Alternatively, the poly(A) addition can be skipped, and/or a different sequence could be used (e.g. poly(C), or s

"For short reference sequences without poly(A)-tails, additional A's or other artificial sequences can be added to the end of th
chimeric reads that involve the very 3' end of the viral genome. If such pads are added, care must be taken when viewing the

4) What is the minimum read size allowed to be considered a valid read for downstream analyses? Are the reads collapsed (in

Similarly as described above, a minimum mappable read length is defined by the fixed-seed length used by bowtie/bwa. To m

There is no explicit support for paired-reads in ViReMa.

'Events' found at the tips of reads are only considered valid if the 'tip' is longer than the required fixed-seed length (i.e. usual

“Additionally, these aligners may be overly permissive in requiring mapped nucleotides on either side of a recombination junc

5) Are the reads clipped above a particular depth coverage? This feature is especially critical in repetitive viral content, such a

No – there is no clipping of reads above a particular depth. ViReMa attempts to map all the reads as exhaustively as possible

6) Have some of these viruses been enriched for targeted capture? Please, provide this information in the manuscript. In som behaves. Moreover, some aligners may have problems in older versions with these depth values.

HIV reads where obtained by sequencing cDNA amplicons derived through targeted RT_PCR of the gag-pol region of HIV. To

“These datasets were obtained by sequencing cDNA amplicons derived from template-specific RT-PCR of the gag-pol genes.”

The SeV samples were not template-enriched.

The STIV samples were obtained from clarified supernatant, as described in the main text, and as such are highly enriched fo

We have corrected the instances of e.g. “300’000” (U.K. format) to “300,000” (U.S. format). This coverage is indeed high, bu input read count would simply down-sample the number of recombination events detected proportionally to the total coverag recombination frequency (i.e. abundance of reads mapped to a recombination junction relative to the number of reads founds

“ViReMa does not make any assumptions with regards to expected read coverage or coverage bias over a reference sequence in the BED files. Recombination ‘frequencies’ can be calculated by comparing the number of reads mapping to a recombinatio target-capture probes and PCR biases result in uneven genome coverage, it is possible that read counts for specific recombin by visualizing the output SAM files in alignment visualization tools such as Tablet (50). “

7) It was unclear which types of duplications were flagged and if the pipeline covers them.

ViReMa can detect and report short duplications in a viral genome. Examples of this are provided in detail in the HIV section, end would be reported as simple recombination events in the BED file, but where the donor site is downstream of the accepto

8) How does the pipeline deal with contaminants?

There is no explicit handling of contaminants by the ViReMa pipeline. Rather, these must be dealt with as appropriate by the removed by aligning to known sources of contamination (e.g. mycoplasma genome). To clarify this point and provide guidanc

“The expected input for ViReMa is FASTQ data from short-read sequencing platforms such as Illumina. Typically, input data s strategies (e.g. using BBDuk from the BBMap suite RRID:SCR_016965) or removing reads that align to known contaminant g

9) This article states that the pipeline works for viral sequences. However, the tests used do not include large genomes. Wha

Thank you for this comment. We have certainly considered the application of ViReMa to larger and more complex genomes ar (as might be present in the boundaries of repetitive elements), additional supporting information is required to fully resolve r area of on-going research that we feel would be beyond the scope of the current manuscript and may indeed illustrate the lin

“We have utilized ViReMa here to uncover evidence of duplications and insertions in clinical HIV isolates in response to antiret junction break-points. However, large duplications that exceed the length of short sequences reads or repetitive regions of sc across complex and variable repetitive elements in vaccinia virus, each containing unique point-mutations (72).“

10) While looking for recombination events, specially fusions with the host, what are the differences between sequenced viral

Thank you for this comment. On a read-by-read sequence information level, there is no way to distinguish between these two Integration events would be reported in the exact same way in ViReMa. This issue is discussed in the final paragraph of the S

“A “Virus-Host-Fusion” event may either describe a direct fusion of viral and host nucleic acids (such as described below for S
whether the input material sequenced derived from the host nucleus) and upon downstream interpretation of the results repo

11) The authors state that the pipeline provides accurate results. Regarding the calculation of accuracy values, several good
a)https://nam11.safelinks.protection.outlook.com/?url=https%3A%2F%2Fwww.sciencedirect.com%2Fscience%2Farticle%2F
MC4wLjAwMDAiLCJQIjoiV2luMzIiLCJBTiI6Ik1haWwiLCJXVCI6Mn0%3D%7C3000%7C%7C%7C&sdata=1fZFx5yLILUdTBaQVbP
b)https://nam11.safelinks.protection.outlook.com/?url=https%3A%2F%2Fwww.sciencedirect.com%2Fscience%2Farticle%2F
MC4wLjAwMDAiLCJQIjoiV2luMzIiLCJBTiI6Ik1haWwiLCJXVCI6Mn0%3D%7C3000%7C%7C%7C&sdata=%2Bfh9KiXKRKYlTDp3H

Thank you for alerting us to these helpful resources.

12) Augmentation of existing pipelines in the area could guide the reader to other solutions and sometimes complementary.
a) ASPIRE: https://nam11.safelinks.protection.outlook.com/?url=https%3A%2F%2Fbmcgenomics.biomedcentral.com%2Fart
8&data=05%7C01%7Calrouth%40utmb.edu%7C975e9cb206e0447b04a808da5b719ff2%7C7bef256d85db4526a72d31aea25
%2Bd70%3D&reserved=0
b) TRACESPipe:
https://nam11.safelinks.protection.outlook.com/?url=https%3A%2F%2Facademic.oup.com%2Fgigascience%2Farticle%2F9%
oiMC4wLjAwMDAiLCJQIjoiV2luMzIiLCJBTiI6Ik1haWwiLCJXVCI6Mn0%3D%7C3000%7C%7C%7C&sdata=t1c7jFHI8UGYgpnCDU
c) V-pipe:
https://nam11.safelinks.protection.outlook.com/?url=https%3A%2F%2Facademic.oup.com%2Fbioinformatics%2Farticle%2F
IjoiMC4wLjAwMDAiLCJQIjoiV2luMzIiLCJBTiI6Ik1haWwiLCJXVCI6Mn0%3D%7C3000%7C%7C%7C&sdata=uep9sRPTxpBijT9bA

Thank you for providing these resources. We have incorporated/references them where appropriate in the main text.

13) Line 113: "in range a of plant" - please correct;

Fixed

14) Line 120-121: Please, rephrase.

Fixed.

15) There are several acronyms; perhaps an abbreviation list would improve the reading of the article.

Added.

16) Line 394: ART is defined as "antiretroviral treated," but this acronym overlaps the ART simulator. Perhaps, in this case, a

Removed instances of ART when referring to HIV treatment and replaced with ‘antiretroviral therapy”

17) Line 753-754: Reference 27 is missing at least the title, journal, and year.

Fixed

18) Please, consider to add ViReMa to Bioconda.

ViReMa has been automatically curated and added to Bioconda:
https://anaconda.org/bioconda/virema

19) I've tried to clone the repository from sourceforge, and it came out empty. I had to download the package manually. I fa
including some tiny read sample and reference along with the code and command description - including how to run the GUI)

For users that wish to use command line access, the sourceforge repository is most simply accessed using the wget comman

wget "https://sourceforge.net/projects/virema/files/ViReMa_0.25/ViReMa_0.25.zip"

A statement to this effect has been added to the methods section for clarification:

"and can be either downloaded manually at this page or by using:
wget "https://sourceforge.net/projects/virema/files/ViReMa_0.25/ViReMa_0.25.zip"'"
As suggested by the reviewer, there is also available some test data, provided in the 'Test_Data' folder that contains a small
which is also displayed automatically when viewing the ViReMa home page in sourceforge.

We have added the following text to emphasize this availability:

"Instructions on how to analyze the small example datasets and to validate correct installation are provided in the associated

Reviewer #2: In this work, Sotcheff et al provide a comprehensive and nicely-written report about using the algorithm Virus
algorithm that, by accounting for the high-diversity nature of virus populations, can efficiently detect a wide range of virus re
advances in NGS, including the read length and the significant increase in NGS library size and NGS-based experiments. Nota
biological connotations. Overall, the paper used a robust analysis method and sufficient controls to clearly demonstrate the ca

We thank the reviewer for their positive assessment and time taken reviewing our manuscript.

Minor comments
1) Since Fig 2E is showing the gradual effect of the permissibility imposed by the error-density values, transforming the table

As suggested – we have generated a barchart to replace the tabulated data in Fig 2E, and adjusted the figure legend accordir

"Bar-chart depicting the number of duplication events using different 'error density' parameters in the command-line. The par

2) At lines 500-501, the author found that the majority of reads mapped directly to the virus genome. Looking at the aligned

We have added the following text to the manuscript:

"Given the large size of the dataset, the default '--Chunk' feature would have processed the reads in packets of 1 million reac

3) At line 478, the authors stated: "The 'Reads' columns describe the number of reads at each particular nucleotide position",

No. This is the exact read count at the coordinates of the referenced template where the unique recombination event has tak

4) Typos at line 206 "red", and at line 397 "(NL4-3)"

Corrected. Thank you!

Close
