# Supplementary material for: ViReMa: a virus recombination mapper of next-generation sequencing data characterizes diverse recombinant viral nucleic acids
Source: Gigascience. 2023 Mar 20;12:giad009. doi: 10.1093/gigascience/giad009 (PMC10025937; doi:10.1093/gigascience/giad009)
Supplement: giad009_Reviewer_1_Report_Original_Submission — Diogo Pratas -- 6/7/2022 Reviewed [file giad009_reviewer_1_report_original_submission.pdf]

Reviewer Report

Title: ViReMa: A Virus Recombination Mapper of Next-Generation Sequencing data characterizes diverse recombinant viral nucleic acids

Version: Original Submission **Date:** 6/7/2022

Reviewer name: Diogo Pratas

Reviewer Comments to Author:

This article describes a pipeline (coded in Python) to detect and analyze recombination events of viral genomes using short-read FASTQ data. The paper presents some level of work accomplished by the authors. Usually, these types of articles hide numerous hours of coding and experimentation. Moreover, the authors present actual accomplishments that typically are unique architectural designs and important alternative ways to the area, including several results. However, many points require attention, namely:1) This pipeline expects exactly a specific virus. Hence, it uses a specific reference. However, this reference might not be the most representative because of the recombination events. Although it may be appropriate for smaller recombination events, detecting large-scale recombinations may face substantial difficulties. Moreover, since it is not prepared to deal with more significant variations (without de-novo support), it is exclusively for targeted support. Therefore, the article could be more descriptive about this specificity.2) The article states that the improvement is also inspired with the read length increase that NGS is bringing. Also, the reported depth coverages are very high. So, why not use de-novo assembly? For example, the de-novo assembly can be used to create scaffolds that can generate a reference sequence to be used after by the aligners. Please, comment on this.3) About the use of artificial poly-(A)tales to allow the mapper to align the reads, what happens when the read size is smaller than the k-mer hash of the aligner? Usually, repetitive A-sequence content appears in almost all samples because they have lower entropy and a higher probability of being generated. Wouldn't this create ambiguity, especially when there are very high-depth coverages? Please, comment on this matter.4) What is the minimum read size allowed to be considered a valid read for downstream analyses? Are the reads collapsed (in the case of Paired-ends) or considered split? Although less probable, the trimming is fundamental for excluding "events" generated at the tips of the reads that very rarely overlap, depending on the nucleotide distribution.5) Are the reads clipped above a particular depth coverage? This feature is especially critical in repetitive viral content, such as hairpins or poly-(A)tales - removing mountains that become the most significant factor in sequence depth coverage.6) Have some of these viruses been enriched for targeted capture? Please, provide this information in the manuscript. In some parts of the article, the coverage depth is very high: 300'000 - is this 300000? The simulated data used this coverage which may not be entirely similar to reality. Also, allowing lower depth coverage helps to understand how the pipeline behaves. Moreover, some aligners may have problems in older versions with these depth values.7) It was unclear which types of duplications were flagged and if the pipeline covers them.8) How does the pipeline deal with contaminants?9) This article states that the pipeline works for viral sequences. However, the tests used do not include large genomes. What about larger genomes? Some larger genomes contain repetitive content that provides

additional reconstruction challenges. Therefore, the benchmark could have an example of this nature.10) While looking for recombination events, specially fusions with the host, what are the differences between sequenced viral integrations and fusion events at the analysis level? How do we distinguish both using this pipeline? Please, comment on this.11) The authors state that the pipeline provides accurate results. Regarding the calculation of accuracy values, several good practices and recommended by many experts in the field:a)https://www.sciencedirect.com/science/article/pii/S1386653220304339b)https://www.sciencedirect.com/science/article/pii/S138665322100079212) Augmentation of existing pipelines in the area could guide the reader to other solutions and sometimes complementary. See, for example:a) ASPIRE: https://bmcgenomics.biomedcentral.com/articles/10.1186/s12864-022-08649-8b) TRACESPipe: https://academic.oup.com/gigascience/article/9/8/giaa086/5894824c) V-pipe: https://academic.oup.com/bioinformatics/article/37/12/1673/610481613) Line 113: "in range a of plant" - please correct;14) Line 120-121: Please, rephrase.15) There are several acronyms; perhaps an abbreviation list would improve the reading of the article.16) Line 394: ART is defined as "antiretroviral treated," but this acronym overlaps the ART simulator. Perhaps, in this case, adding another letter or changing it would remove the ambiguity.17) Line 753-754: Reference 27 is missing at least the title, journal, and year.18) Please, consider to add ViReMa to Bioconda.19) I've tried to clone the repository from sourceforge, and it came out empty. I had to download the package manually. I faced some problems, perhaps because it was not easy to follow. Possibly, users may face the same difficulties, which may be an obstacle to using the software. Please, consider having an elementary example for running ViReMa (already including some tiny read sample and reference along with the code and command description - including how to run the GUI). Please, consider using Github in the following times.

Level of Interest

Please indicate how interesting you found the manuscript: Choose an item.

Quality of Written English

Please indicate the quality of language in the manuscript: Choose an item.

Declaration of Competing Interests

Please complete a declaration of competing interests, considering the following questions:

- Have you in the past five years received reimbursements, fees, funding, or salary from an organisation that may in any way gain or lose financially from the publication of this manuscript, either now or in the future?
- Do you hold any stocks or shares in an organisation that may in any way gain or lose financially from the publication of this manuscript, either now or in the future?

- Do you hold or are you currently applying for any patents relating to the content of the manuscript?
- Have you received reimbursements, fees, funding, or salary from an organization that holds or has applied for patents relating to the content of the manuscript?
- Do you have any other financial competing interests?
- Do you have any non-financial competing interests in relation to this paper?

If you can answer no to all of the above, write 'I declare that I have no competing interests' below. If your reply is yes to any, please give details below.

I declare that I have no competing interests

I agree to the open peer review policy of the journal. I understand that my name will be included on my report to the authors and, if the manuscript is accepted for publication, my named report including any attachments I upload will be posted on the website along with the authors' responses. I agree for my report to be made available under an Open Access Creative Commons CC-BY license (http://creativecommons.org/licenses/by/4.0/). I understand that any comments which I do not wish to be included in my named report can be included as confidential comments to the editors, which will not be published.

Choose an item.

To further support our reviewers, we have joined with Publons, where you can gain additional credit to further highlight your hard work (see: https://publons.com/journal/530/gigascience). On publication of this paper, your review will be automatically added to Publons, you can then choose whether or not to claim your Publons credit. I understand this statement.

Yes Choose an item.
