# Supplementary material for: ViReMa: a virus recombination mapper of next-generation sequencing data characterizes diverse recombinant viral nucleic acids
Source: Gigascience. 2023 Mar 20;12:giad009. doi: 10.1093/gigascience/giad009 (PMC10025937; doi:10.1093/gigascience/giad009)
Supplement: giad009_Reviewer_2_Report_Original_Submission — Fadi G Alnaji -- 7/1/2022 Reviewed [file giad009_reviewer_2_report_original_submission.pdf]

Reviewer Report

Title: ViReMa: A Virus Recombination Mapper of Next-Generation Sequencing data characterizes diverse recombinant viral nucleic acids

Version: Original Submission **Date:** 7/1/2022

Reviewer name: Fadi G Alnaji

Reviewer Comments to Author:

In this work, Sotcheff et al provide a comprehensive and nicely-written report about using the algorithm Virus Recombination Mapper (ViReMa) to identify and characterize different kinds of recombination events in different viruses. ViReMA was first reported - by the same group - in a separate paper (Routh et al, NAR, 2013) as a python-based algorithm that, by accounting for the high-diversity nature of virus populations, can efficiently detect a wide range of virus recombination junctions within virus-derived Next Generations Sequencing (NGS) datasets. In this paper, the authors described a couple of important updates on the original algorithm that enables ViReMa to cope with the new technological advances in NGS, including the read length and the significant increase in NGS library size and NGS-based experiments. Notably, the authors implemented a powerful validation approach by challenging the algorithm with a different type of NGS-based data containing various types of junctions from different viruses to highlight the contextual computational and biological connotations. Overall, the paper used a robust analysis method and sufficient controls to clearly demonstrate the capacity of ViReMa to detect different types of recombinant molecules in different NGS datasets and viruses with high sensitivity and specificity. I only have very few minor comments.Minor comments1) Since Fig 2E is showing the gradual effect of the permissibility imposed by the error-density values, transforming the tables into figures e.g. bar or scatter plots can render the effect more observable visually.2) At lines 500-501, the author found that the majority of reads mapped directly to the virus genome. Looking at the aligned read number, this dataset seems fairly large, I was wondering if using the newly added function --Chunk can come into play at this scenario to speed up the analysis? If it is the case, then maybe mentioning it would be valuable.3) At line 478, the authors stated: "The 'Reads' columns describe the number of reads at each particular nucleotide position", is this the average read number?4) Typos at line 206 "red", and at line 397 "(NL4-3)"

Level of Interest

Please indicate how interesting you found the manuscript: Choose an item.

Quality of Written English

Please indicate the quality of language in the manuscript: Choose an item.

Declaration of Competing Interests

Please complete a declaration of competing interests, considering the following questions:

- Have you in the past five years received reimbursements, fees, funding, or salary from an organisation that may in any way gain or lose financially from the publication of this manuscript, either now or in the future?
- Do you hold any stocks or shares in an organisation that may in any way gain or lose financially from the publication of this manuscript, either now or in the future?
- Do you hold or are you currently applying for any patents relating to the content of the manuscript?
- Have you received reimbursements, fees, funding, or salary from an organization that holds or has applied for patents relating to the content of the manuscript?
- Do you have any other financial competing interests?
- Do you have any non-financial competing interests in relation to this paper?

If you can answer no to all of the above, write 'I declare that I have no competing interests' below. If your reply is yes to any, please give details below.

I declare that I have no competing interests

I agree to the open peer review policy of the journal. I understand that my name will be included on my report to the authors and, if the manuscript is accepted for publication, my named report including any attachments I upload will be posted on the website along with the authors' responses. I agree for my report to be made available under an Open Access Creative Commons CC-BY license (http://creativecommons.org/licenses/by/4.0/). I understand that any comments which I do not wish to be included in my named report can be included as confidential comments to the editors, which will not be published.

Choose an item.

To further support our reviewers, we have joined with Publons, where you can gain additional credit to further highlight your hard work (see: https://publons.com/journal/530/gigascience). On publication of this paper, your review will be automatically added to Publons, you can then choose whether or not to claim your Publons credit. I understand this statement.

Yes Choose an item.
